# Data Query Mechanism Based on Hash Computing Power of Blockchain in Internet of Things

**DOI:** 10.3390/s20010207

**Published:** 2019-12-30

**Authors:** Yongjun Ren, Fujian Zhu, Pradip Kumar Sharma, Tian Wang, Jin Wang, Osama Alfarraj, Amr Tolba

**Affiliations:** 1 School of Computer and Software, Engineering Research Center of Digital Forensics, Ministry of Education, Nanjing University of Information Science & Technology, Nanjing 210044, China; 002315@nuist.edu.cn (Y.R.);; 2 Jiangsu Collaborative Innovation Center of Atmospheric Environment and Equipment Technology (CICAEET), Nanjing University of Information Science & Technology, Nanjing 210044, China; 3 Department of Multimedia Engineering, Dongguk University, Seoul 04620, Korea; 4 College of Computer Science and Technology, Huaqiao University, Xiamen 361021, China; wangtian@hqu.edu.cn; 5 School of Computer & Communication Engineering, Changsha University of Science & Technology, Changsha 410004, China; 6 School of Information Science and Engineering, Fujian University of Technology, Fuzhou 350118, China; 7 Computer Science Department, Community College, King Saud University, Riyadh 11437, Saudi Arabia; atolba@ksu.edu.sa; 8 Mathematics and Computer Science Department, Faculty of Science, Menoufia University, Shebin-El-kom 32511, Egypt

**Keywords:** IoT data, blockchain, cloud storage, hash query

## Abstract

In the IoT (Internet of Things) environment, smart homes, smart grids, and telematics constantly generate data with complex attributes. These data have low heterogeneity and poor interoperability, which brings difficulties to data management and value mining. The promising combination of blockchain and the Internet of things as BCoT (blockchain of things) can solve these problems. This paper introduces an innovative method DCOMB (dual combination Bloom filter) to firstly convert the computational power of bitcoin mining into the computational power of query. Furthermore, this article uses the DCOMB method to build blockchain-based IoT data query model. DCOMB can implement queries only through mining hash calculation. This model combines the data stream of the IoT with the timestamp of the blockchain, improving the interoperability of data and the versatility of the IoT database system. The experiment results show that the random reading performance of DCOMB query is higher than that of COMB (combination Bloom filter), and the error rate of DCOMB is lower. Meanwhile, both DCOMB and COMB query performance are better than MySQL (My Structured Query Language).

## 1. Introduction

The concept of the IoT (Internet of Things) has been proposed for a long time, and a large amount of practical applications have also come into practice. The IoT has a wide range of applications in many industries, such as logistics, manufacturing, and so on [1,2,3]. With the popularization and application of the IoT, big data, and intelligent manufacturing have naturally emerged. IoT applications retrieve massive data to form large datasets [4,5,6]. By mining these data, useful decision information is obtained to support the manufacturing industry upgrade. IoT data can mine information for decision making and the optimization of production processes, but because of the challenges of the IoT data system itself, (1) heterogeneity of IoT system results, along with the (2) resource constraints of IoT devices, and (3) privacy and security vulnerability [7,8,9]. Thus, the use of existing IoT data is still at a lower level.

The increasingly popular blockchain technology can make up for some of the existing shortcomings of the IoT. Blockchain is essentially an open and transparent distributed general ledger. The information on the blockchain is traceable and tamper-proof, which means that the information is trustworthy [10,11,12]. However, this also causes problems. Encrypted information can be placed directly on the blockchain and will be disclosed. This will reveal the confidentiality of important information and cause it to lose value. The most direct application of the existing blockchain is the cryptocurrency. Taking bitcoin as an example [13], the bitcoin mining process can be considered as a miner machine that has been doing hash operations. The average bit time of bitcoin is about 10 min, which is adjusted by the “Mining difficulty adjustment algorithm”. This also means that the more computing power, the greater the difficulty and the lower the energy efficiency of the miners. Recently, the existing ASIC (Application Specific Integrated Circuit) mining machine can only do hash calculations. In the future, few bitcoins remain unexplored. When the mining difficulty is too large, a lot of hash calculation machines will be withdrawn. How to reuse these idle computing power will be the research hotspot. Therefore, there must be a lot of research on the conversion of hash computing power in the future [14].

The blockchain and the IoT also share similarities. The blockchain is a general ledger across the distributed system. All nodes on the IoT system can be regarded as distributed nodes of the system. This similarity also provides a basis for technology convergence. Blockchain is a particularly perfect complement to the IoT, which can be used to improve the interoperability, security, privacy, reliability, etc. of IoT systems [15,16,17]. In this case, BCoT was proposed to create a safer, more reliable, and more versatile technology by combining blockchain and IoT. More importantly, the intelligent contract of the blockchain provides an incentive mechanism to ensure the high scalability of the database, as well as reward and punishment for the honesty of the node [18,19,20]. The efficient processing efficiency of smart contracts facilitates the construction of fully automated data processing processes.

The roadmap of this paper is organized as follows. Section 2 introduces the knowledge of IoT technology, blockchain, smart contract, and BCoT. Section 3 presents the status and analysis of the problem. In Section 4, we will introduce the IoT data query model based on blockchain. Section 5 will explain our proposed power conversion method for the data query. Then, experiments and results analysis will be explained in Section 6. Finally, the conclusion is given in Section 7.

## 2. Related Work

### 2.1. IoT Technology

The earliest idea of the IoT was to add RFID (Radio Frequency Identification) and other information sensing devices to all items to connect to the Internet for online identification and management [21,22]. In short, the IoT is the technology that connects objects to the network, enabling the integration of the entire physical world. This technology has developed rapidly in recent years, and even the trend of the IoT is ubiquitous. The large-scale application of the IoT has also produced a huge amount of data, which is valuable but needs to be analyzed. Analyze the data first to analyze the characteristics of the data. IoT data has many unique attributes:

1. The data is chronological, so there must be a timestamp.
2. There are very few updates and deletes for data
3. The user is concerned with the trend for some time, not the value of a specific point in time.
4. Massive data.

The vast amount of data of the IoT is intelligently processed and analyzed. Finally, the data model is obtained and fed back to the decision-making system to form a smart IoT [23]. Therefore, the data of the IoT will become more and more important in the future.

### 2.2. Blockchain

The emergence of blockchain technology is slowly affecting other existing information and communication technologies [24,25,26,27,28]. Its application can help overcome the shortcomings of existing technologies. The blockchain is a distributed general ledger constructed on the P2P (Peer-to-Peer) network using a consensus mechanism between nodes. Blockchains also naturally provide distributed timestamp services. It uses timestamps to implement a time-ordered chain of blocks. Each new block is time-stamped and finally connected into a blockchain according to the order in which the blocks are generated. Each independent node establishes a connection through the P2P network, thus forming a record for the information data. Centralized distributed timestamp service system.

#### 2.2.1. Structure of Blockchain

In general, a blockchain is a distributed general ledger with timestamps built on a P2P network using a consensus mechanism between nodes [29,30]. Blockchain has the characteristics of de-neutralization, anonymity, traceability, transparency, and temper-proof.

**Figure 1:**
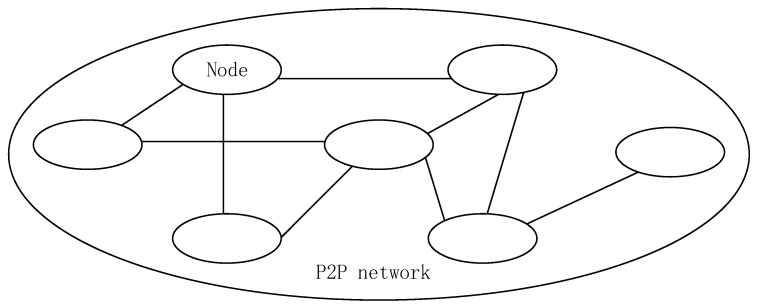
The structure of the P2P network.

The blockchain is built on the P2P network and exists in every node in the P2P network. The P2P network applied in the blockchain is a computer network that is peer-to-peer, decentralized, and distributed. In a P2P network, all nodes have equal status and functions. The shared resources set by the nodes, such as computing resources, storage space resources, and network resources, can be shared by other nodes. The more nodes that are added to this network, the more resources are shared, and the better the service quality of the entire system. The decentralized nature of the P2P network brings it scalability and robustness, which also lays the foundation for the success of the blockchain. The P2P network structure is shown in Figure 1.

**Figure 2:**
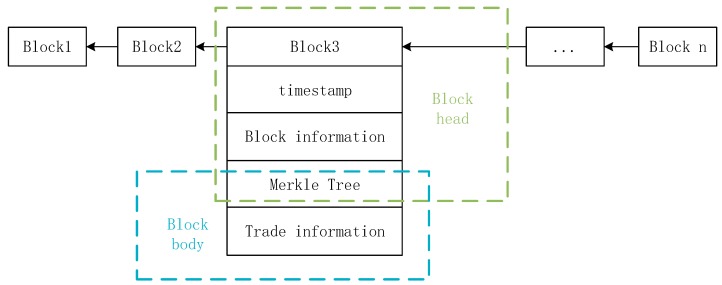
The basic structure of the blockchain.

Blockchain is exemplified by bitcoin. The first block is called the Genesis block, and each block can be divided into a block head and a block body. The block header stores the timestamp, block information, and Merkle tree. There is trading information in the block. The block information is mainly the address of the previous block, the random number, the timestamp, the target hash value of the current block, and the root of the Merkle tree, wherein the timestamp is the block time of the block. The block mainly stores transaction information. The hash of all transaction information constitutes the Merkle tree, and the root of the Merkle tree is placed in the block header. The Merkle tree is cleverly designed to quickly verify the integrity and authenticity of your data. This also greatly improved the transaction efficiency and scalability of bitcoin. The structure of the blockchain is shown in Figure 2.

#### 2.2.2. Bitcoin Mining

Bitcoin mining is to collect and confirm the transaction data that has not been confirmed by the network since the last block, and then package it into a transaction block that cannot be tampered with, so as to complete a network recognized transaction record and keep it for a long time. The mining process is to find the x that makes the following formula true.

SHA256(SHA256(version+prev_hash+merkle_root+ntime+nbits+x))<TARGET

The range of x above is 0 to 2^32^, and TARGET can be calculated based on the current difficulty. According to the design of the bitcoin system, there are only 21 million bitcoins in the system. A transaction block can be produced every 10 min and the reward for bitcoin will be halved every four years until the mining reward is complete. Difficulty adjustment will ensure the block time is around 10 min. In 2019, the maximum hashing rate of the whole bitcoin network is 114 EH/s. The computational power of a Nvidia 1080Ti is approximately 75 MH/s, so the total net computational power is approximately $1.52*10^{12}$ 1080 Ti graphics cards.

### 2.3. Smart Contract

A smart contract is a digital contract that exists on the blockchain and is ready to execute [31,32]. It allows us to perform retroactive and irreversible and secure protocols without the need for a trusted third party. A smart contract contains all the information about the transaction and will only execute the contract if it meets the requirements. Smart contracts are more efficient and less secure. The advantage of using smart contracts is that they are more efficient, irreversible, and secure when dealing with transactions. On the other hand, the shortcoming is the lack of legal supervision, human error, and implementation. There have also been instances of contract losses leading to losses in Ethereum. Finally, a smart contact provides an incentive mechanism to ensure the high scalability of the database and reward and punishment for the honesty of the node. The efficient processing efficiency of smart contracts facilitates the construction of fully automated processes.

### 2.4. BCoT

BCoT is blockchain with IoT [33,34]. Blockchain has many unique merits, such as transparency, non-reputation, tamper proof, traceability. Blockchain is a nearly perfect complement to IoT, which can improve the interoperability, privacy, security and scalability of IoT. Moreover, more and more IoT applications such as smart homes, digital factories combined with blockchain, have achieved good results. Blockchain technology helps intelligent manufacturing improve data interoperability across production departments through P2P networks and production-related data security can also be guaranteed [35,36]. In the food production industry, the integration of blockchain and the production supply chain can provide producers and consumers with tamper-proof and traceable production and sales information, which not only ensures food safety but also consumers can eat with confidence [37,38,39,40]. The integration of health care and blockchain can overcome the security and privacy protection of medical health data [41,42,43]. Blockchains are also used in smart grids [44,45,46], supply chain management [47], and the Internet of Vehicles [48,49,50], and this is enough to see a wide range of applications for blockchain applications.

## 3. Problem Statement

In this section, we summarize some of the existing problems of the IoT and blockchain and analyze them.

Traditional IoT systems have many challenges:

• Data heterogeneity

Different IoT structures vary widely, and the types of data and data they receive vary widely. Therefore, different structures also cause different environments, and it is impossible to apply an application in the IoT directly to another IoT.

• Node resource constraints

The hardware resources of IoT devices are limited in terms of computing power, storage resources, network performance, and battery energy. The low computational performance of IoT nodes for encryption and decryption requires most of the computing resources and battery power. Nodes have limited storage capacity and cannot store large amounts of data. Communication capabilities are generally not maintained for long periods and the communication rate is slow due to battery capacity. Some long-term online nodes are connected to the power supply, so battery power is not considered.

• Security vulnerable

The distributed structure and heterogeneity of IoT systems make it difficult to ensure the security of the entire system, but security is the most basic requirement for an application system. But for lightweight nodes with limited resources, encryption and decryption are very resource-intensive. The impact of malicious node attacks on the system is also not negligible.

There are also some problems in the blockchain, such as the problem of bitcoin computing power conversion. As mentioned above, bitcoin mining is mainly for miners to do hash calculations. Now, there is a huge amount of hash computing power that will become a huge waste when the unexploited bitcoin is almost exhausted in the future, and most of the existing miners are ASIC machines. The hash operation is not universally calculated, so how to use these computing powers in the future is a promising research direction.

In response to the above questions, we made some analyses.

Blockchain technology can help many of the existing problems of the IoT. The intrinsic time stamping function of the blockchain can replace the timestamp of IoT data, thus simplifying the data structure of the IoT. Further, the traceability of the blockchain can be combined with database query operations to enable IoT data to support traceable queries. In addition, the blockchain’s non-destructive modification can help to verify the integrity of the encrypted data.

(a) Using the concept of data flow, the structure of IoT data is reduced from small to large.

IoT data can be divided into two categories: one is static data, which is generally the attribute of the sensor nodes and the data of the tag class, and their number increases with the increase of the IoT sensor nodes. The second type is dynamic data, which is the data generated by sensor nodes, which increases with time. According to these two characteristics, the data generated by one sensor can be aggregated into one data stream. Like the current, the water flows continuously from the source to form a river. Static data is the source of the data stream, and dynamic data is the source of the data. When constructing a data set, you only need to construct a K-S data structure. K is a static data key which is the keyword of the data stream and S is a dynamic data stream. When you perform data operations, you only need to find the key to find the data stream. Simplify the IoT data structure through data flow, making data-related operations more concise.

(b) Extend the storage of the IoT with blockchains and smart contracts, replacing the timestamp of the original data with the timestamp function of the blockchain. Dynamic data are generated with its timestamp. The time attribute is a very important attribute of IoT data. The IoT can use the timestamp function of the blockchain technology to eliminate the time stamp of dynamic data. It can omit storage space and speed up the query.

(c) The encrypted data index is written into the blockchain, and the encrypted data is uploaded to the cloud storage server to ensure that the data is not leaked, and the integrity of the data and whether it has been tampered with can be verified. Encrypted data can also be searched, and data structures that support hardware and distributed structure searches can quickly query the data stream.

(d) The main hash operation is mainly to convert the target text into an irreversible hash message digest of the same length, which is applied to the data structure and the password. When used in the data structure, it is mainly to improve the efficiency of the query. This pays more attention to the calculation and verification speed of the target hash value. It is not worthy of confrontation collision, as long as the hash is evenly distributed. In cryptography, the hash algorithm is mainly used for message digest and signature. It can only verify the integrity of the message, and cannot encrypt and decrypt the message.

## 4. IoT Data Query Model based on Blockchain

### 4.1. System Mode-Blockchain-Based IoT Data Query Model

**Figure 3:**
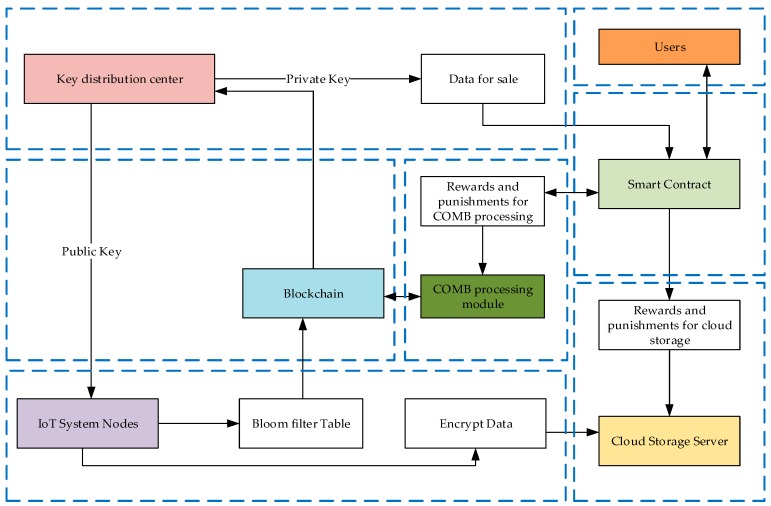
Blockchain-based IoT data model.

This paper designs a blockchain-based IoT data query model as shown in Figure 3. In this data model, the data query is performed by the hash of the data stream header, and the query first checks the timestamp to find the block where the data of the required period is located and then searches in the block. This model consists mainly of the public key and the private key distribution modules, blockchains, smart contracts, COMB processing modules, IoT systems, cloud storage servers, and users. The blockchain generates the Genesis block as a signal to start the operation of the entire model. Next, each block generated by the blockchain sends a signal to the key distribution center to distribute the public key-private key pair. The key distribution center sends the encrypted public key to the IoT. The public key is used to encrypt IoT data, and the public and private keys are packaged as sales data for transmission to smart contracts. The nodes in the IoT system need to first send you the public key to the key distribution. After obtaining the public key, send the encrypted data to the cloud storage server, and send the data header hash, the encrypted data hash and the public key to the blockchain. The COMB processing module directly reads the data on the blockchain and constructs the COMB for the query. Smart contracts reward and punish the work of key distribution centers, COMB processing modules, and cloud storage servers. When a user wants to get data, he only needs to purchase it from a smart contract, and the smart contract returns the encrypted data and its corresponding private key.

The role of these modules is described separately below.

- Blockchain: The blockchains are chronologically ordered and the blocks are equally spaced. Each block stores the data header hash sent by the IoT node, encrypting the data hash and the public key. After the data is uploaded to the blockchain, it has the advantage of being traceable and tamper-proof.
- Key distribution: Generate a public-private key pair that is sent to the IoT node to encrypt the data. The public and private key pairs are packaged into the sales data and sent to the smart contract.
- Smart contract: Trading data with users, rewarding and punishing the storage of cloud storage servers and the operation of COMB processing modules
- IoT system: The IoT nodes collect data, encrypts the data with the received public key, sends the encrypted data to the cloud storage server, and sends the data stream header hash, the encrypted data hash and the public key to the blockchain. Therefore, after the node uploads the data, all the data is encrypted, and the query operation is performed in the encrypted data environment. This also increases the security of the data.
- The cloud storage part: This part stores the encrypted data sent by the IoT node; cooperates with the smart contract to expand the storage space of the IoT node.
- COMB processing part: This part gets the data header hash-public key pair from the blockchain, distributed parallel construction data query table, and distributed parallel query. The operations in the module are all hash operations, so you can call the mining machine’s hash power.
- User Part: Trading to a smart contract to get the data you want.

### 4.2. Cloud-Blockchain Model

The goal of constructing this data structure is to query the data using only hash operations, and the data on the blockchain is public and transparent, so the data uploaded to the block must be encrypted. Also, the data uploaded to the block should not be too large, which is not conducive to the efficiency of data processing. According to the above analysis, the data structure needs to have the following characteristics

- The data must be encrypted before being uploaded to the block.
- The data uploaded by the node is concise, that is, the index, and the complete encrypted data can be placed on the cloud server.
- The data uploaded to the blockchain is deterministic and cannot be tampered with.
- Data query is in the environment of data encryption and is searched by encrypting data index.
- The best data operation uses hash computing power.

According to the above analysis, we use the data stream hash, the data stream ciphertext hash and the public key as an index of data retrieval. Through the data stream header, the data stream ciphertext hash and public key can be queried. The private key can be detected by the corresponding public key, and the data stream ciphertext is requested by the data stream ciphertext hash.Finally, the private key is used to decrypt the data stream ciphertext to obtain the data plaintext. Each block has data for a specified time. When the user needs data for multiple periods, it is necessary to query multiple blocks.

**Figure 4:**
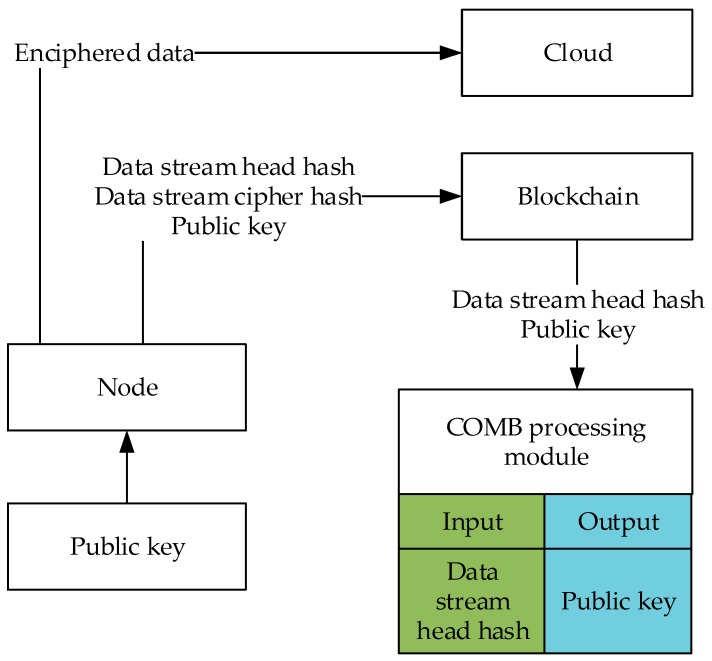
IoT node data processing flow.

The flow of processing data by the IoT node is shown in Figure 4. First, the node requests the public key from the key distribution center and then uses this public key to encrypt the data during this time. The encrypted data is then sent to the cloud storage server. The data stream head hash, data stream cipher hash, and public key are sent to the block. The data query requires the COMB processing module to construct a lookup table and then query. Enter the stream header hash for the COMB processing module, which will return the encrypted public key for this data.

### 4.3. Data Storage Model

**Figure 5:**
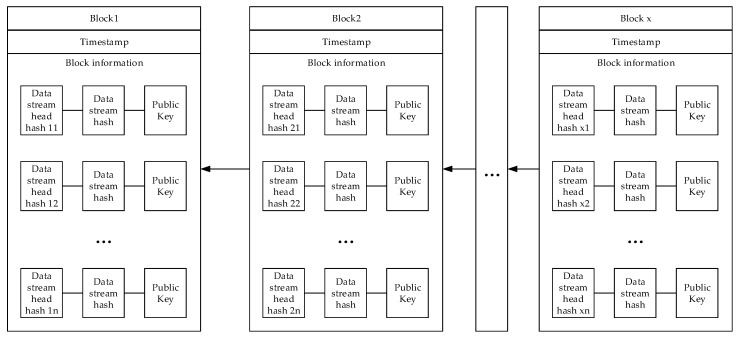
The structure of the data storage model based on the blockchain.

The structure of the data storage model based on the blockchain is shown in Figure 5. First, the blockchain is composed of blocks, each block has a timestamp. Although the node cannot upload the current data at the time of the block generation, it can be artificially specified. The node generates the interval between the block generation time and the node uploading data so that the accurate node data generation time can be obtained by using the block generation time minus the interval time.

The information in the node existence block is the data stream header hash, the data stream hash, and the public key. Both the stream header hash and the stream hash are summary messages, and the original text of the message cannot be restored by the digest message, which is determined by the irreversible nature of the hash function. These data are encrypted, which requires the query operation to be performed in an encrypted environment. This also increases the security of IoT data. Although all the data on the block is publicly transparent, these are encrypted data. For users without a private key, it is useful to obtain a summary of the information and the encrypted information. The data uploaded by the other nodes is simple, and it takes up very little storage space compared with the plaintext of the data. This also speeds up the query efficiency of the entire data model.

With the help of blockchain technology, all data uploaded to the node is open and transparent, and tamper-proof. This is provided by the nature of the blockchain. The encrypted data in the cloud storage server can verify the integrity of the data through the data hash on the blockchain. Any machine can download the data summary in the block and operate, which also lays the foundation for multiple machines to process the data in parallel. Each block only stores the data uploaded by the IoT node from the time of generation of this block to the next block. When a user needs to check multiple blocks of data for multiple periods, these blocks are checked separately, and these check operations can be performed simultaneously.

## 5. Data Query Scheme

**Figure 6:**
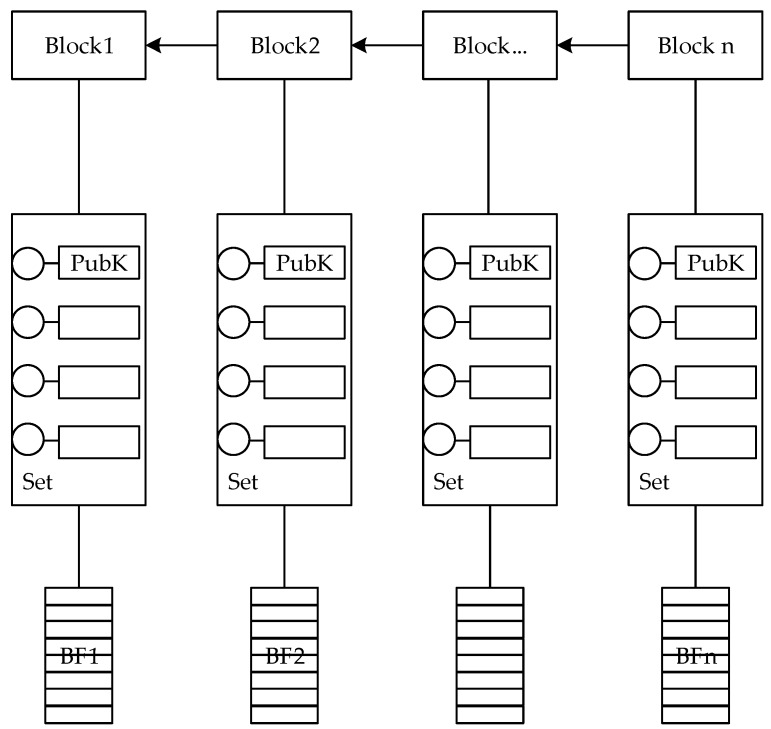
The data query scheme.

The data query scheme we designed is shown in Figure 6. The scheme is to query the corresponding public key through the data stream hash. Firstly, we need to make a BF (Bloom filter) for all the stream headers in each block, to quickly query whether there is our data in this block. Next, make a DCOMB (dual combinatorial Bloom filter) for all the data in each block. In the structure of DCOMB, the public key is used as the id, and the data stream header is used as the index element. The DCOMB processing module of the entire system can return the index element of the public key through the input.

According to the characteristics that the IoT data does not decrease over time, this data structure does not exist for deletion and update operations. After the node’s data is uploaded to the blockchain, the data is irreversible and traceable. The DCOMB module reads the data disclosed by the blockchain and constructs the BF and DCOMB. When the data is queried, the check operation can be performed to obtain the public key of the data stream. However, because of the false positive from the hash collision, position misjudgment can be caused. This false positive rate can be estimated and controlled. Besides, all query operations of this data structure use hash computing power and support distributed parallel operations. All query operations can be queried in the case of data encryption. Finally, the query operation in this scheme only requires hash computing power to complete.

### 5.1. Preliminary Knowledge

Before we introduce this solution in detail, we need to explain some preliminary knowledge.

#### 5.1.1. Bloom Filter

The Bloom filter is a probabilistic data structure for efficient insertion and query [51,52]. It can return that an element may or may not exist. The Bloom filter consists of a very long binary vector and a series of hash mapping functions. The use of the Bloom filter requires first recording a set of n elements, $E=\left\{e_{0},\;e_{1},\;..\;e_{n-1}\right\}$ in the vector. This insertion operation is called encoding. After all the inserts have been completed, you can ask if an element is present in the Bloom filter. This operation is called checking.

The Bloom filter has a wrong answer which called a false positive. A false positive is that there is a certain probability that an element that does not exist is judged to exist, which is caused by a hash collision. All data structures consisting of Bloom filters have such problems. The probability of false positive is described below. The Bloom filter has an m-bit binary vector. All bits of the initial vector are 0 and k independent hash functions.

The r-th element is denoted as $e_{r}$, (0≤r≤n−1).

The i-th element in the Array is denoted as B[i], (0≤i≤m−1).

The j-th and hash functions are denoted as $hash_{j}$, (0≤j≤k−1).

In the insert operation, insert the r-th element in the i position of the array as

(1) B[i]=B[hj(er)]=1,for(0≤j≤k−1)

In the query operation, each hash function of the query element returns a value, which is denoted as $B\left[h_{j}\left(e\right)\right]$. If all m hash functions return $B\left[h_{j}\left(e\right)\right]=1$, then e may exist in the array that has a large positive. If the return value of a hash function is not 1, then e must not exist in the array. False positive is to give a positive answer to a non-existent element error. Pf is the false positive rate in the Bloom filter. Further, Pf is the smallest when

(2) $$k=\frac{m}{n}\times \ln2.$$

The Bloom filter can only detect if an element is in an array and does not tell us where the element is. Since all elements are known to exist and are not added, delete and update dynamic operations, so the impact of dynamic updates is not considered.

#### 5.1.2. DCOMB and COMB

DCOMB (dual combinatorial Bloom filter) consists of two layers of COMB (combinatorial Bloom filter). COMB is a novel Bloom filter-based data structure that supports multi-set membership test [53,54,55,56]. And can be implemented in hardware. COMB meets time-critical web applications. COMB can not only answer an element that is not in the collection but also return the specific position of the element in the collection. COMB is to maintain the elements along with the associated groups in the dynamic data structure that permit fast classification.

COMB construction: S represents a set of n elements, where each element belongs to one of y Groups. G stands for these y groups. From x elements in S, g(x)∈G indicates that x is in G, and g(x) can also be considered as the number of x in G, which is denoted as $G_{id}$. Enter x in COMB, if x is output to g(x) in S or an empty set if x is not in S. So, all legal output is G∪{∅}.

A group number l, l∈G, use l to represent the number of x in S, so g(x)=l.

COMB has three error types:

- False normal: The false normal here is the same as in the Bloom filter, and the element that does not exist is judged to be normal, which is a false normal.
- Misclassification: Misclassification means that COMB gives some x∈S the wrong group id which still belongs to G∪{∅}.
- Classification failed: Classification failure does not return the wrong group id but cannot return the result. The result does not belong to G∪{∅}.

Evaluate COMB performance indicators:

- COMB capacity: In short, the storage capacity occupied by COMB.
- Insert operation memory read times: The number of memory read accesses required to insert all elements.
- Checking operation memory read times: The number of memory read accesses required to query an element.

The hash function is not the bottleneck in a COMB [55].

### 5.2. Block Data Query Scheme Based on Bloom Filter and Dual COMB

**Figure 7:**
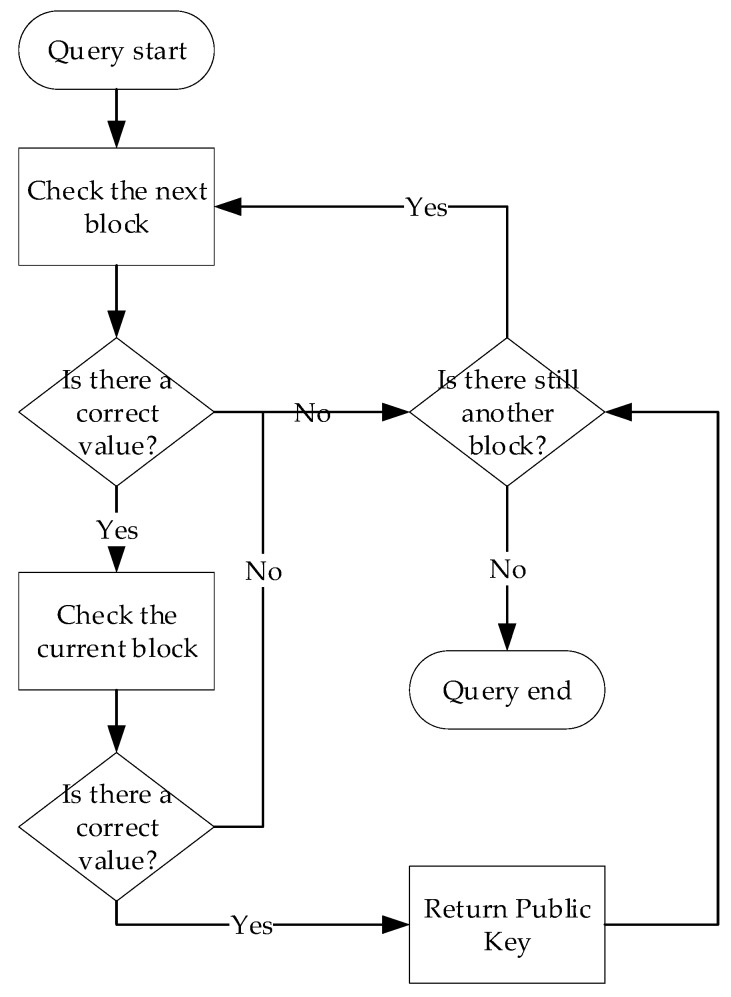
The Data in the blockchain query process.

Block data query requires two steps. First, build a Bloom filter for all query indexes in each block, and then build a DCOMB in each block. Finally, the query operation can quickly query the correct information in the encryption environment by using the two-level query table, and only a small possibility may return an error result, which will be analyzed later. The function of the Bloom filter is to check if there is any relevant information in the block. The Bloom filter will return no and possibly. If there is a possibility of a correct value in the block, a DCOMB query is required. Then, the correct value will be returned, and the error value will be returned with a small probability. The query process is shown in Figure 7.

#### 5.2.1. Bloom Filter Query for All Blocks

After the IoT node data index is uploaded to the blockchain, it cannot be tampered with. So, the current time can’t change the previous data index, which means that the previous blocks except the latest block cannot be updated, and the rest of the block data is determined. Then, when constructing the Bloom filter, all parameters can be adjusted according to the number of data indexes in the block.

The criterion for parameter selection is to minimize the false positive probability. False positive means Bloom filter incorrectly gives a positive answer to a member query. For a block $B_{i}$ from the set of all blocks $B=\{B_{1},B_{2},B_{3},\ldots ,B_{i},\ldots ,B_{s}\}$, and suppose the length of the Bloom filter is $m_{i}$, a total of $n_{i}$ indexes and $k_{i}$ hash functions. According to the actual situation that $n_{i}$ has been determined and the parameters of $m_{i}$ and $k_{i}$ are appropriately adjusted to minimize the false positive probability. False positive probability represented by $Pf_{i}$, can be calculated as

(3) $$Pf_{i}={\left(1-{\left(1-\frac{1}{m_{i}}\right)}^{n_{i}\times k_{i}}\right)}^{k_{i}}\approx {\left(1-e^{-\frac{n_{i}\times k_{i}}{m_{i}}}\right)}^{k_{i}}$$

where $Pf_{i}$ is minimized when

(4) $$k_{i}=\frac{m_{i}}{n_{i}}\times \ln2.$$

The Bloom filter has two main operations, encoding and checking. The role of encoding is to write all the indexes in each block to the Bloom filter corresponding to the block. The role of checking is to check the Bloom filter of each block to see if there is a correct value.

(a). Encoding

Bloom filter $BF_{i}$ for block $B_{i}$ is an array of $m_{i}$ bits whose initial values are all 0. The l-th bit is represented by $BF_{i}\left[l\right]$, $0\leq l\leq m_{i}-1$. There are $n_{i}$ index elements in the block. The r-th element is denoted as $e_{r}$, $0\leq r\leq n_{i}-1$. There are $k_{i}$ hash mapping functions. The j-th and hash functions are denoted as $h_{j}$, $0\leq j\leq k_{i}$.

If the r-th index element will be inserted into the filter, and the operation is to map the element to the filter with $k_{i}$ hash mapping functions. The encoding operation of the j-th hash function is to map the position to the filter to 1. Inserting an element into the Bloom filter $BF_{i}$ with a hash mapping function compute as

(5) $$BF_{i}\left[h_{j}\left(e_{r}\right)\right]=BF_{i}\left[l\right]=1.$$

Each element of the block is mapped to all the $k_{i}$ hashes on the bloom filter $BF_{i}$, so that the encoding is complete. Inserting all element completely into a Bloom filter with all the hash mapping functions compute as

(6) $$BF_{i}\left[h_{j}\left(e_{r}\right)\right]=BF_{i}\left[l\right]=1.\;0\leq j\leq k_{i}-1,\;0\leq r\leq n_{i}-1.$$

The elements of all blocks are inserted into their Bloom filter. The total number of hash operations to be spent is as follows.

(7) $${Num}_{\mathrm{hash}\;\mathrm{operations}}=\sum_{B}\sum_{n_{i},i\in B}k_{i}$$

(b). Checking

Since our solution is to store the data in the block corresponding to the period, the correct data may exist in each block. The Bloom filter of all blocks is queried, and further queries are required if the correct values are possible. For a hash query index element y, every Bloom filter of the block will be checked to confirm if it exists. Query in one block $B_{i}$ requires all the relevant hash mapping functions of this block to calculate all positions of y on the filter and check if these positions are 1. This query operation is calculated as

(8) $$BF_{i}\left[y\right]=BF_{i}\left[h_{j}\left(y\right)\right],\;0\leq j\leq k-1.$$

If one of these locations, $h_{j}\left(y\right)$ is not 1, then y cannot be in this block. If these locations are all 1, then y is in this block with a certain probability which is 1−pf. This means that this block requires a more detailed query.

For an index that needs to be queried, all the blocks need to be queried, and the number of hash calculations required for the query operation is as follows.

(9) $$Num_{hash}=\sum_{B}k_{i}$$

(c). False positive probability of the first level query

There are s blocks in total, and the Bloom filters of each block are independent, and they all have their false normal probability.

(10) $$Pf\left(all\;BF\right)=Pf\left(BF_{1}\right)\cup Pf\left(BF_{2}\right)\cup Pf\left(BF_{3}\right)\cup \ldots \cup Pf\left(BF_{i}\right)\cup \ldots \cup Pf\left(BF_{s}\right)$$

So, the total false normal probability at the first level of the query is as follows.

(11) $$pf\left(all\;BF\right)=\sum_{B,i\in B}Pf_{i}$$

#### 5.2.2. DCOMB for Query

**Figure 8:**
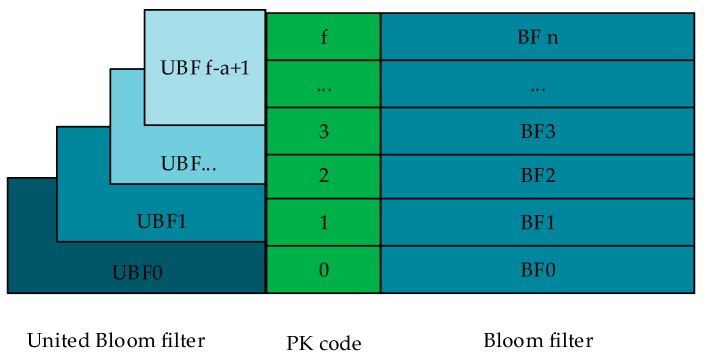
The Dual COMB structure.

After the block query, the blocks that returned the correct value has been confirmed. Next, the correct index values will be looked up in these blocks. The DCOMB structure is shown in Figure 8, and its structure is two-layer combination Bloom Filter. First, the public key is converted into a binary array which is called PK code. On the left is a union-bits UBF (United Bloom filter), and on the right is the BF (Bloom filter) for each bit. When asking for an element, first check the UBF on the left. If it returns 1, check the separate BF on the right. The advantage of this is that the write access is increased in exchange for the read access reduction and the query is accelerated.

A set of binary arrays transformed by public keys is called PK code. All public keys in a block need to be converted to PK code, and the length of these PK codes are the same, a total of f+1 bits. f+1 is the number of bits of the longest PK code. For public keys less than f+1 bits, the remaining bits are supplemented with 0. Dual COMB needs to construct two layers of COMB, the first layer consists of UBF and PK code. The second layer consists of BF and PK codes. These two layers will be described in detail below.

(1). First COMB with UBFs

The first COMB starts with the PK code from the lowest bit, and each a bit is combined and called union bits. Each united bit corresponds to a union Bloom filter called UBF. There are n index elements in each block, UBF is m bits long, and $\frac{h}{a}$ hash mapping functions are used in each UBF. If the PK code of an element is 0 in this union bits, then the element is not inserted into the UBF of the union bits; if an element’s PK code has a bit in the union bits that is not 0, then the element is inserted into the UBF of the union bits.

(a). Insertion

Suppose an index y in block $B_{i}$ needs to be inserted. The binary PK code converted by the public key corresponding to y has f+1 bits. Starting from the lowest bit, each a bit constructs a union bits, then all joint bits are represented by U, $U=\left\{u_{0},\;u_{1},.\;.\;.\;\;u_{f-a+1}\right\}$. Each union bits corresponds to a UBF. All union bits with individual bits that are not 0 need to write the index y to the corresponding UBF. According to the index to be written, the union bits to be written are also different, and the ratio of the union bits u which will be written to all the union bits is $\theta_{y}$. The insert operation of y is to insert the index y into all union bits that are not zero. The sum of the memory access for insert index *y* is

(12) $$Num_{memory\;access\;for\;inserting\;y}=U\times \theta_{y}\times \frac{h}{a}$$

The memory access required to insert all the indexes in the block is

(13) $$Num_{memory\;access\;for\;inserting\;all}=\sum_{n}U\times \theta_{y}\times \frac{h}{a}$$

(b). Checking

Suppose block $B_{i}$ needs further query for PK code of index y. The purpose of this query is to get rough information about PK code with as little memory access as possible. This requires querying all UBFs in the block $B_{i}$. The sum of the memory access for query the PK code of index *y* is

(14) $$Num_{memory\;access\;for\;querying\;y}=U\times \frac{h}{a}$$

The memory access required to query all the PK codes of indexes in all blocks is

(15) $$Num_{memory\;access\;for\;querying\;all}=\sum_{n}U\times \frac{h}{a}$$

Finally, the union bits with a single bit of 0 will be returned. These bits need further query for more precise PK code.

The first COMB returns the union bits that do not exist. The possibility of this non-existence is 100%, which does not need to be discussed. But each UBF has a false normal probability, and the sum of all UBF false normal probabilities in a block is

(16) Pf(all UBFs in a block)=Pf(UBF0)+Pf(UBF0)+…+Pf(UBF f−a+1)

Each UBF has a different number of index elements, so when constructing UBF, all elements are used, n. The other parameters are UBFs with the lowest false normal probability constructed with n as a reference. And each UBF is m bits long, has n parameters, uses $\frac{h}{a}$ hash functions, and their false normal probability is theoretically the same. All UBFs do not use all these elements. According to the formula, when n becomes small, the false positive probability becomes low. So, the UBF constructed from the number *n* of all elements will be higher than the actual false positive probability. The false positive probability of all UBFs in a block is

(17) $$Pf\left(all\;UBFs\;in\;a\;block\right)>\left(f-a+2\right)\times {\left(1-e^{-\frac{n\times \frac{h}{a}}{m}}\right)}^{\frac{h}{a}}$$

$\frac{h}{a}$ is the number of hash functions and must be a number more than 1, so this probability is still very low.

(2). Second COMB with BFs

After the first COMB query, all separate bits that need to be carefully queried are determined. The second COMB is to construct a BF for each bit to query the exact PK code. Each BF has m bits, and there are n index elements, which need to use h hash functions. The second COMB needs to consider all the individual bits when inserting, meanwhile the bits other than 0 are inserted into the index element. And all index elements are inserted into COMB. Only individual bits which not excluded by the first COMB need to be queried. The bits which return inexistence in the first COMB will not be queried here.

(a). Insertion

Suppose an index y in block $B_{i}$ needs to be inserted. The binary PK code converted by the public key corresponding to y has f+1 bits. Only the BF corresponding to the 1 bit needs to be inserted into the index y. Assume that the proportion of bits in the PK code of the element is η. The ratio of the index y is $\eta_{y}$. For index y, all the memory access required for the insertion is $h\times \eta_{y}\times \left(f+1\right)$. The memory access required to insert all the elements is

(18) $${Num}_{\mathrm{memory}\;\mathrm{access}\;\mathrm{required}\;\mathrm{to}\;\mathrm{insert}}\sum_{n}h\times \eta_{n}\times \left(f+1\right)$$

(b). Checking

The second COMB check is not the same as the traditional COMB check, because the first COMB has been filtered, so there is no need to check all the bits. In the first COMB, the ratio of the union bits that are not 0 is θ, and these bits need to be further checked. Suppose the ratio of these bits is λ. Then the number of bits that need to be checked is (f+1)×λ. The memory access required to insert an index y is (f+1)×λ×h. Then, the memory access required to check all the elements in a block is

(19) $${Num}_{\mathrm{memory}\;\mathrm{access}\;\mathrm{required}\;\mathrm{to}\;\mathrm{check}}=\sum_{n}\left(f+1\right)\times \lambda_{n}\times h$$

(c). Computing Error Probability

The calculation error is an error when returning a bit in the PK code, resulting in the final PK code not corresponding. That is because a certain bit of BF returned 1 wrongly. Let Pce denote the probability of this bit being wrong, μ denotes the ratio of the element to be written in this bit to the total n index element, and m is the length of the Bloom filter corresponding to this bit. m,h are the best parameters chosen according to n with the smallest false positive probability. Then, the false positive probability of this Bloom filter is

(20) $$\begin{array}{cc} Pce & ={\left(1-{\left(1-\frac{1}{m}\right)}^{\mu nh}\right)}^{h}\\ & \approx {\left(1-e^{-\frac{\mu nh}{m}}\right)}^{h} \end{array}$$

(d). False Positive probability

Now the PK code has an f+1 bit length, where q bit exactly already has a false positive, and each BF has a false positive probability of p, so the false positive probability of all BFs is

(21) $$\begin{array}{cc} \mathrm{Pr}\left(False\;positive\;probability\right) & =p^{q}\times {\left(1-p\right)}^{f-q+2}\\ & \leq p^{q} \end{array}$$

q is greater than 1, so the pseudo-normal probability of the remaining bits is also very low.

(e). Classification Failure Probability

If one of the (f−q+2)BFs incorrectly returns 1 when we make the second COMB query, classification failure will occur.

(22) $$\begin{array}{cc} \mathrm{Pr}\left[Classification\;Failure\right] & =1-{\left(1-p\right)}^{f-q+2}\\ & \leq \left(f+1-q\right)p\\ & \leq fp \end{array}$$

The possibility of classification failure is generally much greater than the probability of false positive. If they are almost one order of magnitude, the memory size needs to be adjusted.

DCOMB has more union bits than COMB, so DCOMB needs more storage space and more memory access. The DCOMB of the union bits has the same number of bits and the same number of elements as the COMB of the individual bits. The number of hash mapping functions is different, and the number of BF hash function is a times the UBF. The parameter of BF is a Bloom filter of the smallest false normal probability constructed with the number n of indices. Therefore, the false normal probability of UBF will be higher, but the probability is also small. Also, the UBF query is a bit that does not exist and returns a bit that requires further query. The Bloom filter returns an element that does not exist, so it cannot exist. Therefore, the false positive command of UBF affects the need for multiple bits of multiple queries and does not affect the result. The joint bit of COMB will greatly help reduce the query and ultimately speed up the output of the query results.

#### 5.2.3. Distributed Parallel Processing Method

**Figure 9:**
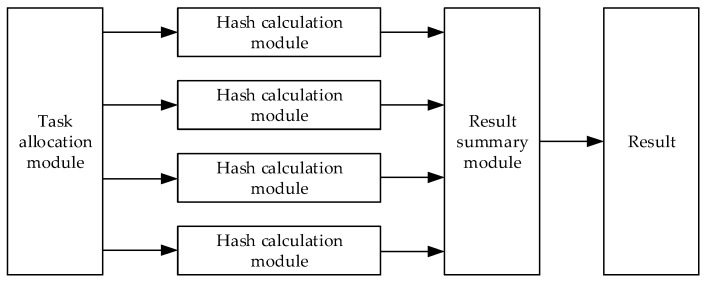
Distributed parallel processing method.

This entire data query structure can be distributed and parallel processed, and its concept is shown in Figure 9. Because each step of the query uses hash operation, the task allocation module can assign the hash operation required by the current query to all the hash calculation modules, and each module completes the data and sends the result to the structure summary module. Finally, this module returns the correct result after processing the summary results.

## 6. Experiment and Result Analysis

In this section, DCOMB will be evaluated by simulation. We will confirm that the performance of DCOMB in the query compared to the best available scheme COMB in terms of random read, error rate and worst and universal performance in continuous reading and writing. Our experimental platform is a computer with intel i3 3.3GHz CPU, 8GB of memory, and 120GB SSD. The software environment is Windows 10, IntelliJ IDEA 2019, java 1.8.0_20, and MySQL8.0.

We used the same parameters in the DCOMB and COMB Bloom filters and selected the same sample database which has 103,976 data during testing. Each COMB has 20 Bloom filters, each Bloom filter has $2^{24}$ bits, which occupies 2MB space and has 8 hash functions. So, both false positive probability and classification failure probability should not be greater than $10^{-7}$. Moreover, the first COMB has 18 Bloom filters which have three hash functions and 18 Bloom filters, and the second COMB has 20 Bloom filters which are the same with COMB.

**Figure 10:**
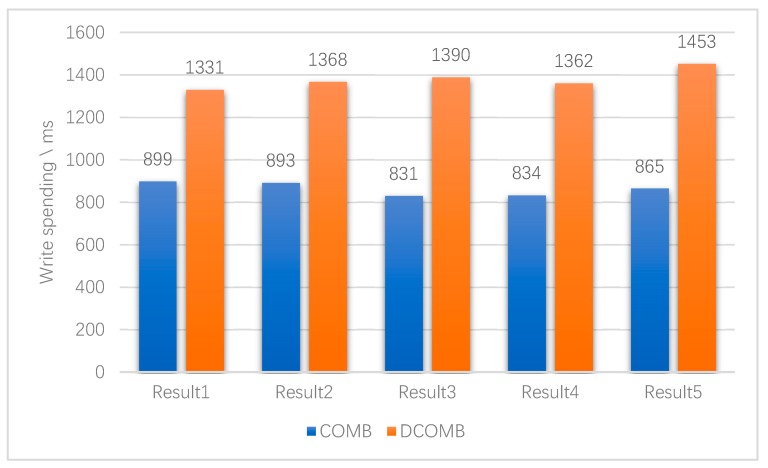
Time-consuming to insert data.

The first experiment is to compare the time it takes for DCOMB and COMB to query all the data indexes. The results of five experiments are shown in Figure 10. DCOMB generally takes about half as much time to insert all data than COMB. This is in line with DCOMB theory. DCOMB requires an additional 18 UBFs and each UBF has three hash functions. So, inserting an index into DCOMB requires nearly half of insert memory access than COMB.

**Figure 11:**
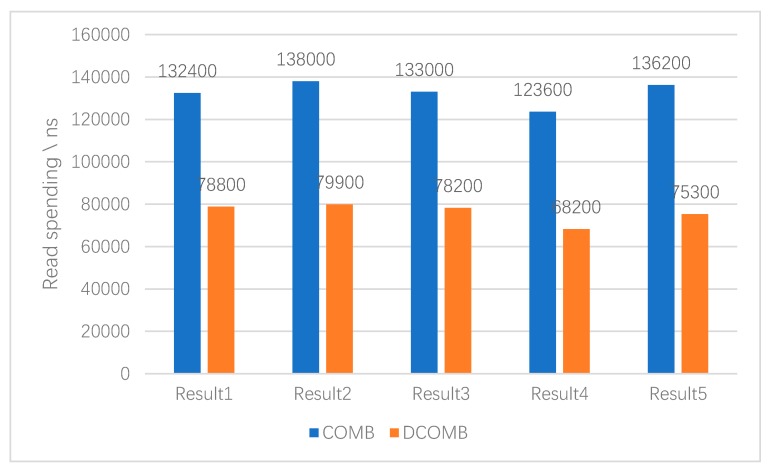
Random read time-consuming.

The second experiment tests random read performance. In the experiment, DCOMB and COMB read some randomly selected data indexes. The results are shown in Figure 11 that DCOMB takes less time to read the data index randomly than COMB. This is because the first COMB in DCOMB requires less check memory access when reading data than COMB. Although this pre-read takes additional time, this result will get the approximate location of the index, which can also be used as a reference for the second DOMB.

**Figure 12:**
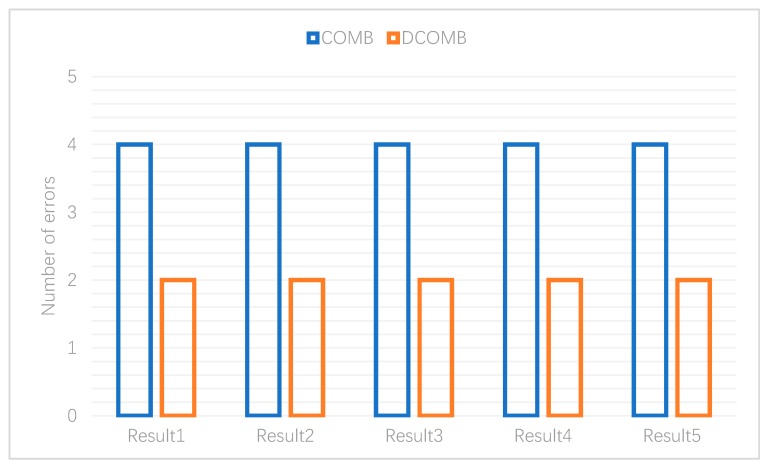
Number of errors.

The third experiment is to detect the classification failure of DCOMB and COMB. This verifies all the inserted indexes and gets the number of errors. The results are shown in Figure 12 that the number of DCOMB errors is lower than that of COMB. When querying the first layer of COMB of DCOMB, all UBFs are verified, and the bits that UBF returns to be non-existent will be set to 0. According to the nature of the Bloom filter, the correct rate of Bloom filter returning non-existence is 100%. Therefore, this layer of structure will first determine the possible false positive bits as 0, and does not verify the bit which is 0 during the second layer COMB verification. So, DCOMB also has a lower probability of error than COMB.

**Figure 13:**
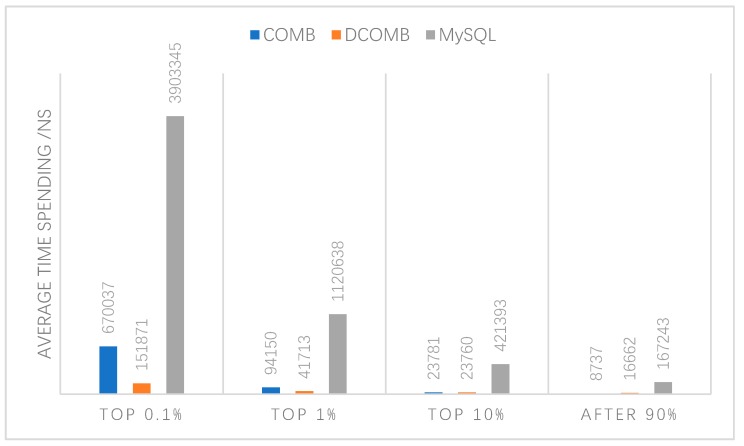
COMB, DCOMB and MySQL comparison.

The last experiment was to query all the data using COMB, DCOMB, and MySQL query methods. The query time of each data will be sorted from high to low. The worst performance measures the stability of a query method. The results are shown in Figure 13 that among the 0.1% of data with the longest query time, the average time of DCOMB is the lowest, while that of MySQL is the highest and of 90% of data with the shortest query time, the average time of COMB is the lowest, and MySQL is the highest. The structure of COMB is simpler than DCOMB, so it has advantages in continuous query. The two-tier COMB of the DCOMB method is mainly to simplify the second COMB query. Therefore, DCOMB’s random read and write speed is also faster, which was reflected in the second experiment. In short, the worst query of the DCOMB method will take less time than COMB. These two queries have many advantages over ordinary MySQL.

The results of the above experiments show that it is feasible to query data with hash computing power, and the query error rate is extremely low. Further, the query speed is much faster than ordinary queries. COMB’s continuous read query is fast, DCOMB’s random read query is faster, and the worst query result of DCOMB is better than COMB. DCOMB also has less classification failure. DCOMB and COMB query methods are much faster than ordinary MySQL queries.

## 7. Conclusions

In this paper, we propose a query method called DCOMB (dual combination Bloom filter), which converts hash computing power of bitcoin mining into computing power of query innovatively. Compared with the existing hash query methods of COMB, the extra union-bits COMB in our scheme can improve query performance. Experimental results show that both DCOMB and COMB perform much better than MySQL, and DCOMB has higher random read query performance, lower worst query performance, and a lower error rate than COMB. Further, our query model also supports distributed parallel processing. The more hash machines participate in the query, the higher the efficiency of the model. In the future, when the untapped resources of bitcoin are scarce, the idle mining computing power can be effectively utilized.

This article also proposes a blockchain-based IoT data query model based on the DCOMB query method, using the concept of data flow instead of the traditional K-V data structure. Replacing the time attribute of IoT data with the blockchain timestamp can make the data more concise and improve query efficiency. This scheme encrypted the node data and uploaded it to the cloud storage server to solve the problem of insufficient storage space for IoT nodes. Smart contracts automatically reward and punish the entire data model, promoting a virtuous cycle of data utilization. The data query model in this paper can quickly query the public key corresponding to the data stream. The query is in a fully encrypted environment, which can ensure the privacy and security of IoT data.
